# Oral Administration of *Lacticaseibacillus rhamnosus* CRL1505 Modulates Lung Innate Immune Response against *Klebsiella pneumoniae* ST25

**DOI:** 10.3390/microorganisms11051148

**Published:** 2023-04-28

**Authors:** Stefania Dentice Maidana, Yoshiya Imamura, Mariano Elean, Leonardo Albarracín, Keita Nishiyama, Yoshihito Suda, Shoichiro Kurata, María Ángela Jure, Haruki Kitazawa, Julio Villena

**Affiliations:** 1 Laboratory of Immunobiotechnology, Reference Centre for Lactobacilli (CERELA-CONICET), Tucuman 4000, Argentina; stefi.dentice@gmail.com (S.D.M.); melean@cerela.org.ar (M.E.); lalbarracin@herrera.unt.edu.ar (L.A.); 2 Laboratory of Antimicrobials, Institute of Microbiology “Luis C. Verna”, Faculty of Biochemistry, Chemistry and Pharmacy, National University of Tucuman, Tucuman 4000, Argentina; 3 Food and Feed Immunology Group, Laboratory of Animal Food Function, Graduate School of Agricultural Science, Tohoku University, Sendai 981-8555, Japan; yoshiya.imamura.p8@dc.tohoku.ac.jp (Y.I.); keita.nishiyama.a6@tohoku.ac.jp (K.N.); 4 Livestock Immunology Unit, International Education and Research Centre for Food and Agricultural Immunology (CFAI), Graduate School of Agricultural Science, Tohoku University, Sendai 981-8555, Japan; 5 Department of Food, Agriculture and Environment, Miyagi University, Sendai 980-8572, Japan; suda@myu.ac.jp; 6 Laboratory of Molecular Genetics, Graduate School of Pharmaceutical Sciences, Tohoku University, Sendai 980-8578, Japan; shoichiro.kurata.d5@tohoku.ac.jp

**Keywords:** *Lcb. rhamnosus* CRL1505, *K. pneumoniae*, innate immunity, probiotics

## Abstract

Orally administered *Lacticaseibacillus rhamnosus* CRL1505 enhances respiratory immunity, providing protection against respiratory viruses and *Streptococcus pneumoniae*. However, the capacity of the CRL1505 strain to improve respiratory immunity against Gram-negative bacterial infections has not been evaluated before. The aim of this work was to evaluate whether the *Lcb. rhamnosus* CRL1505 was able to beneficially regulate the respiratory innate immune response and enhance the resistance to hypermucoviscous KPC-2-producing *Klebsiella pneumoniae* of the sequence type 25 (ST25). BALB/c mice were treated with the CRL1505 strain via the oral route and then nasally challenged with *K. pneumoniae* ST25 strains LABACER 01 or LABACER 27. Bacterial cell counts, lung injuries and the respiratory and systemic innate immune responses were evaluated after the bacterial infection. The results showed that *K. pneumoniae* ST25 strains increased the levels of TNF-α, IL-1β, IL-6, IFN-γ, IL-17, KC and MPC-1 in the respiratory tract and blood, as well as the numbers of BAL neutrophils and macrophages. Mice treated with *Lcb. rhamnosus* CRL1505 had significantly lower *K. pneumoniae* counts in their lungs, as well as reduced levels of inflammatory cells, cytokines and chemokines in the respiratory tract and blood when compared to infected controls. Furthermore, higher levels of the regulatory cytokines IL-10 and IL-27 were found in the respiratory tract and blood of CRL1505-treated mice than controls. These results suggest that the ability of *Lcb. rhamnosus* CRL1505 to help with the control of detrimental inflammation in lungs during *K. pneumoniae* infection would be a key feature to improve the resistance to this pathogen. Although further mechanistic studies are necessary, *Lcb. rhamnosus* CRL1505 can be proposed as a candidate to improve patients’ protection against hypermucoviscous KPC-2-producing strains belonging to the ST25, which is endemic in the hospitals of our region.

## 1. Introduction

Hypervirulent *Klebsiella pneumoniae* has become one of the most relevant pathogens in the world. The alarming emergence of this pathogen is due to the wide dissemination of different variants with the increasing ability to resist several antibiotic treatments, including carbapenems. In fact, carbapenemase-producing *K. pneumoniae* strains have been associated with a wide range of hospital acquired-infections and are associated with high morbidity and mortality rates [1,2,3]. In recent years, an increase in the prevalence of hypermucoviscous carbapenem-resistant *K. pneumoniae* of the sequence type (ST) 25 was detected in patients hospitalized in Northwest Argentina. This hypermucoviscous phenotype has been associated with hypervirulent strains, leading this bacterium to cause different infectious diseases including liver abscesses, meningitis, endophthalmitis, prostate abscesses and respiratory infections [4,5]. In this sense, our previous studies have shown that *K. pneumoniae* carbapenemase-2 (KPC-2)-producing ST25 strains are endemic in hospitals in the province of Tucuman (Argentina) and are associated with respiratory and systemic infections [5,6,7]. Notably, hypermucoviscous KPC-2-producing *K. pneumoniae* ST25 strains are resistant to almost all available antibiotics, making the search for strategies to prevent their infections a high priority.

Probiotic microorganisms have been proved to improve the resistance to pathogens. Using in vitro approaches or mice models of multiresistant *K. pneumoniae* intestinal colonization, it was demonstrated that some *Lactobacillus* spp. strains diminished the pathogen colonization and biofilm formation [8,9,10,11,12]. The ability of lactobacilli to inhibit the colonization of *K. pneumoniae* was associated with organic acid production [10,11] and the secretion of proteins with antimicrobial activity [13,14]. In addition, few studies reported that the oral administration of probiotic microorganisms to mice significantly reduced the lung inflammatory damage induced by *K. pneumoniae* nasal challenge [15,16], demonstrating that immunomodulatory bacteria are an interesting alternative to enhance resistance to this pathogen.

*Lacticaseibacillus rhamnosus* CRL1505 has been widely studied in our laboratory because of its remarkable immunomodulatory properties. Our previous reports demonstrated that the nasal priming with *Lcb. rhamnosus* CRL1505 enhanced respiratory immunity, improving protection against respiratory syncytial virus (RSV), influenza virus (IFV) and *Streptococcus pneumoniae* [17,18]. We also demonstrated that orally administered CRL1505 strain is able to beneficially modulate innate and adaptive immune responses in the respiratory tract, allowing a reduction of the susceptibility to viral and Gram-positive bacterial infections [19,20,21,22]. However, the capacity of orally administered *Lcb. rhamnosus* CRL1505 to modulate respiratory immunity in the context of Gram-negative bacterial infections in general, and multiresistant pathogens in particular, has not been evaluated before. Thus, the aim of this work was to evaluate whether the immunomodulatory strain *Lbc. rhamnosus* CRL1505 was able to beneficially regulate the respiratory innate immune response and enhance the resistance to hypermucoviscous KPC-2-producing *K. pneumoniae* ST25.

## 2. Materials and Methods

### 2.1. Microorganisms

*Lacticaseibacillus rhamnosus* CRL1505 belong to the CERELA (Reference Center for Lactobacilli, CONICET, Tucuman, Argentina) Culture Collection. The CRL1505 strain was isolated from goat milk [17,18]. *Lcb. rhamnosus* was cultured for 18 h at 37 °C, the late log phase, in MRS broth (Man–Rogosa–Sharpe, Britannia). After growth, bacteria were harvested by centrifugation at 3000× *g* for 10 min, washed three times with sterile 0.01 M phosphate-buffered saline (PBS, pH 7.2) and suspended in sterile PBS.

LABACER 01 and LABACER 27, hypermucoviscous carbapenem resistant *K. pneumoniae* ST25 strains, were isolated at the “Angel Cruz Padilla” hospital (San Miguel de Tucuman, Tucuman, Argentina). ST25 *K. pneumoniae* strains were identified by MALDI-TOF (matrix-assisted laser desorption/ionization) (Microflex LT; Bruker Daltonik GmbH, Bremen, Germany) and kept in the Culture Collection of the Certified Bacteriology Laboratory (LABACER, National University of Tucuman, Tucuman, Argentina) [4,5,6].

### 2.2. Animals

Six-week-old male BALB/c mice were obtained from the closed colony maintained in the CERELA-CONICET (Tucuman, Argentina) Animal Facility. All procedures for handling animals were performed according with the guide for the care and use of laboratory animals and approved by the CERELA-CONICET Animal Care and Ethics Committee under the BIOT-CRL/19 protocol. Eighteen mice were divided into three groups: basal, *K. pneumoniae* LABACER 01 (Kp01) and *K. pneumoniae* LABACER 27 (Kp27). Each of these groups was further subdivided into two groups. Half were treated with *Lcb. rhamnosus* CRL1505 while the other half received no treatment. Thus, each subgroup contained 3 mice and the experiments were repeated three times (n = 9 for each parameter evaluated). During the experiments, mice were housed in plastic cages at room temperature and fed a conventional balanced diet ad libitum.

*Lcb. rhamnosus* CRL1505 was administered via the oral route in a dose of 10^8^ cells per mouse per day during 5 consecutive days. The dose was selected as optimal for immunomodulation in previous studies [17,18]. On day 6, the administration of the CRL1505 strain was terminated and mice were challenged with *K. pneumoniae* strains, as described below. During the following 2 days post-infection mice did not receive the CRL1505 strain.

### 2.3. Respiratory Infection with K. pneumoniae LABACER 01 and LABACER 27

*K. pneumoniae* LABACER 01 or LABACER 27 were administered to lightly anesthetized mice dropwise, through the nostrils. For this purpose, 100 uL of sterile PBS containing 10^7^ CFU of bacteria were used. Mice in the control group were administered only 100 µL from PBS. The infective dose selected for this work was determined previously [23,24].

### 2.4. K. pneumoniae Counts in Lungs and Blood

Bacterial cell counts in the lungs and blood were performed in mice sacrificed on day 2 post-infection. The lungs of animals were excised, weighed, and homogenized in 5 mL of sterile peptone water. Homogenates were appropriately diluted in BHI broth, plated on blood agar and cultured at 37 °C for 18 h. *K. pneumoniae* LABACER 01 or LABACER 27 colonies were counted, and results were expressed as log_10_ CFU per gram of lung. Hemocultures were performed similarly using 100 µL of blood, and results were expressed as log_10_ CFU per mL of blood.

### 2.5. Lung Damage

Broncho alveolar lavages (BAL) were used for the determination of albumin and lactate dehydrogenase (LDH) [17,20]. BAL samples were obtained according to the technique described previously [13]. The trachea was exposed and intubated with a catheter, then two sequential bronchoalveolar lavages were performed in each mouse by injecting sterile PBS. The recovered fluid was centrifuged for 10 min at 900× *g* and the fluid frozen at −70 °C for subsequent determinations. Albumin, as a measure to quantify the increased permeability of the alveolar–capillary barrier, was determined colorimetrically based on albumin binding to bromocresol green using a Wiener Lab albumin diagnostic kit. LDH activity, an indicator of cellular cytotoxicity, was determined by measuring the formation of the reduced form of NAD^+^ using Wiener’s reagents and procedures. Results were expressed as units per liter of BAL.

### 2.6. Leukocytes Counts

The total number of leukocytes and differential cell counts in BAL samples were determined as described previously [13,14]. A hemocytometer was used to determine the total number of BAL and blood leukocytes. Differential cell counts in BAL and blood were assessed by microscopically counting cells in smears stained with May–Grunwald–Giemsa.

### 2.7. Serum Cytokines and Bronchoalveolar Lavages

BAL samples were obtained as described above. Blood samples were obtained via cardiac puncture and collected in heparinized tubes [14,15]. Cytokines determinations were performed in serum and BAL samples using commercial ELISA kits: IFN-γ (Mouse IFN-gamma Quantikine ELISA Kit, sensitivity: 2 pg/mL), and IL-10 (Mouse IL-10 Quantikine ELISA Kit, sensitivity: 5.2 pg/mL) from R&D Systems (Minneapolis, MN, USA), TNF-α (Mouse TNF alpha ELISA Kit, sensitivity: 9.1 pg/mL), IL-27 (Mouse IL-27 p28/IL30 Quantikine ELISA Kit, sensitivity: 4.7 pg/mL), IL-17 (Mouse IL-17 Quantikine ELISA Kit, sensitivity 5 pg/mL), IL-6 (Mouse IL-6 Quantikine ELISA Kit, sensitivity: 1.8 pg/mL), IL-8 mouse homolog chemokine KC (Mouse CXCL1/KC DuoSet ELISA, sensitivity 2.3 pg/mL), IL-1β (Mouse IL-1 beta ELISA Kit (ab197742), sensitivity: 1 pg/mL) and MCP-1 (Mouse MCP1 ELISA Kit (ab208979), sensitivity: 0.487 pg/mL) from Abcam (Cambridge, UK).

### 2.8. Statistical Analysis

The experiments were performed in triplicate and the results were expressed as mean ± SD. Statistical analyses were performed using Prism 8.0 (GraphPad software). Comparisons among multiple groups across multiple time points were performed using a two-way ANOVA with Tukey’s multiple comparison post hoc test. Comparisons between two groups were performed using unpaired Student’s *t*-tests. Differences were considered significant at *p* < 0.05.

## 3. Results

### 3.1. Lcb. rhamnosus CRL1505 Improves Resistance to K. pneumoniae ST25 Infection

**Figure 1:**
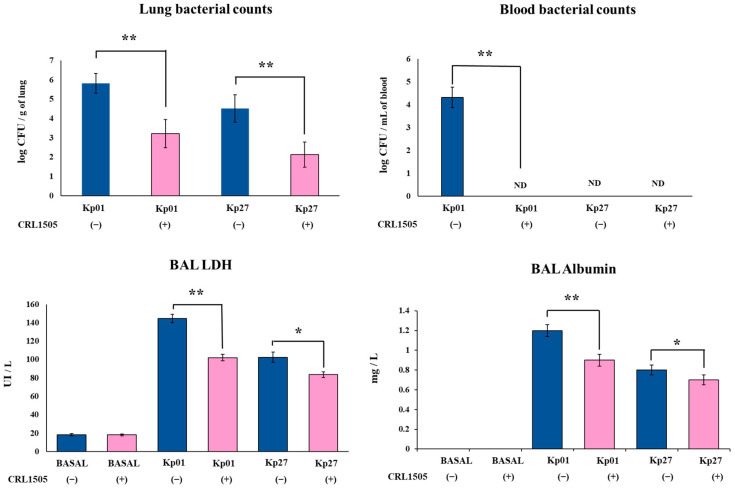
Effect of *Lacticaseibacillus rhamnosus* CRL1505 on lung colonization and damage induced by KPC-2-producing hypermucoviscous *Klebsiella pneumoniae* ST25 strains. BALB/c mice (6 weeks old) were orally treated with the CRL1505 strain for 5 days and then challenged nasally with *K. pneumoniae* LABACER 01 (Kp01) or LABACER 27 (Kp27). Before (basal) and two days after challenge, bacteria cell counts in lung homogenates, broncho–alveolar lavages (BAL) lactate dehydrogenase (LDH) activity and albumin concentration were determined. Results represent data from three independent experiments. Asterisks indicate significant differences between the indicated groups, (*) *p* < 0.05, (**) *p* < 0.01. Basal levels of BAL albumin were below the detection limit. ND: not detected. Pink bars represent mice given *Lcb. rhamnosus* CRL1505 and blue bars represent control mice.

*K. pneumoniae* strains were able to colonize the respiratory tract of mice, with the LABACER 01 strain (5.8 ± 0.5 log CFU/g of lung) being more efficient than LABACER 27 strain (4.5 ± 0.6 log CFU/g of lung) (Figure 1). In addition, blood bacterial cultures showed that only *K. pneumoniae* LABACER 01 was able to spread from the lungs (4.3 ± 0.4 log CFU/mL of blood), since the blood cultures in the LABACER 27 group were negative (Figure 1). The oral treatment of mice with *Lcb. rhamnosus* CRL1505 before the challenge with the *K. pneumoniae* ST25 strains significantly reduced lung bacterial cell counts (3.2 ± 0.6 and 2.1 ± 0.6 log CFU/g of lung for LABACER 01 and LABACER 27, respectively) (Figure 1). The treatment with the CRL1505 strain was also capable of avoiding the dissemination of *K. pneumoniae* LABACER 01 into blood (Figure 1).

The biochemical markers albumin and LDH were studied in BAL samples to evaluate the lung damage induced by the KPC-2-producing hypermucoviscous *K. pneumoniae* ST25 strains. In normal conditions, albumin is not detected in BAL samples, hence the increase in this parameter indicates an enhanced permeability of the bronchoalveolar–capillarity barrier. In addition, the increase in the intracellular enzyme LDH in BAL samples is an indicator of cytotoxicity. As shown in Figure 1, both LABACER 01 and LABACER 27 strains significantly increased BAL albumin (1.21 ± 0.06 and 0.87 ± 0.05 mg/L, respectively) and LDH levels (144.5 ± 4.5 and 102.5 ± 5.6 UI/L, respectively) compared to non-infected controls. It was also observed that mice orally treated with *Lcb. rhamnosus* CRL1505 prior the infection with *K. pneumoniae* ST25 strains had significantly lower levels of BAL albumin (0.91 ± 0.06 and 0.71 ± 0.04 mg/L for LABACER 01 and LABACER 27, respectively) and LDH (102.1 ± 3.5 and 83.7 ± 3.1 UI/L for LABACER 01 and LABACER 27, respectively) than those found in their respective control groups (Figure 1). 

### 3.2. Lcb. rhamnosus CRL1505 Modulates Innate Immunity against K. pneumoniae ST25 Infection

**Figure 2:**
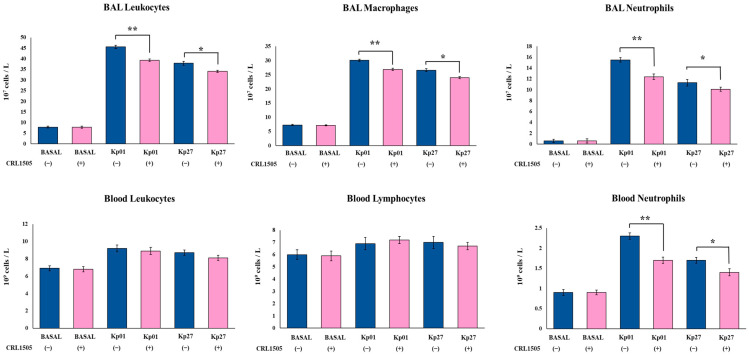
Effect of *Lacticaseibacillus rhamnosus* CRL1505 on respiratory and blood leukocytes numbers induced by KPC-2-producing hypermucoviscous *Klebsiella pneumoniae* ST25 strains. BALB/c mice (6 weeks old) were orally treated with the CRL1505 strain for 5 days and then challenged nasally with *K. pneumoniae* LABACER 01 (Kp01) or LABACER 27 (Kp27). Before (basal) and two days after challenge, total and differential leukocytes counts were determined in broncho–alveolar lavages (BAL) and blood. Results represent data from three independent experiments. Asterisks indicate significant differences between the indicated groups, (*) *p* < 0.05, (**) *p* < 0.01. Pink bars represent mice given *Lcb. rhamnosus* CRL1505 and blue bars represent control mice.

We further studied the influence of the CRL1505 strain on immune cells in both respiratory and systemic compartments (Figure 2). The oral treatment with *Lcb. rhamnosus* CRL1505 did not modify the numbers either of BAL or blood leucocytes in the steady state. The respiratory challenge with *K. pneumoniae* ST25 strains significantly increased the numbers of BAL leucocytes, macrophages and neutrophils in both control and lactobacilli-treated mice (Figure 2). However, mice treated with the CRL1505 strain showed statistically reduced levels of BAL leucocytes, macrophages, and neutrophils compared to controls. In fact, BAL leucocyte counts in control mice infected with LABACER 01 and LABACER 27 strains were 45.6 ± 0.8 and 37.9 ± 0.9 10^7^ cells/L while in mice treated with the CRL1505 strain they were 39.3 ± 0.6 and 34.1 ± 0.5, respectively.

Similarly, the respiratory infection with LABACER 01 and LABACER 27 enhanced the levels of blood leukocytes, neutrophils, and lymphocytes (Figure 2). No significant differences were found between control and CRL1505-treated mice after the challenge with *K. pneumoniae* LABACER 01 or LABACER 27 when blood leukocytes and lymphocytes were compared. In contrast, significantly lower numbers of blood neutrophils were observed in mice orally treated with *Lcb. rhamnosus* CRL1505 after infection with both LABACER 01 and LABACER 27 strains (Figure 2). Blood neutrophil counts in control mice infected with LABACER 01 and LABACER 27 strains were 2.31 ± 0.08 and 1.72 ± 0.07 10^7^ cells/L, while in mice treated with the CRL1505 strain they were 1.71 ± 0.06 and 1.43 ± 0.06, respectively.

**Figure 3:**
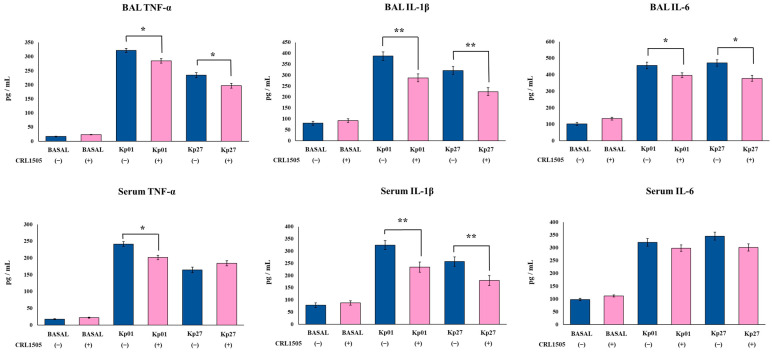
Effect of *Lacticaseibacillus rhamnosus* CRL1505 on respiratory and blood inflammatory cytokines induced by KPC-2-producing hypermucoviscous *Klebsiella pneumoniae* ST25 strains. BALB/c mice (6 weeks old) were orally treated with the CRL1505 strain for 5 days and then challenged nasally with *K. pneumoniae* LABACER 01 (Kp01) or LABACER 27 (Kp27). Before (basal) and two days after challenge, TNF-α, IL-1β and IL-6 levels were determined in broncho–alveolar lavages (BAL) and serum. Results represent data from three independent experiments. Asterisks indicate significant differences between the indicated groups, (*) *p* < 0.05, (**) *p* < 0.01. Pink bars represent mice given *Lcb. rhamnosus* CRL1505 and blue bars represent control mice.

**Figure 4:**
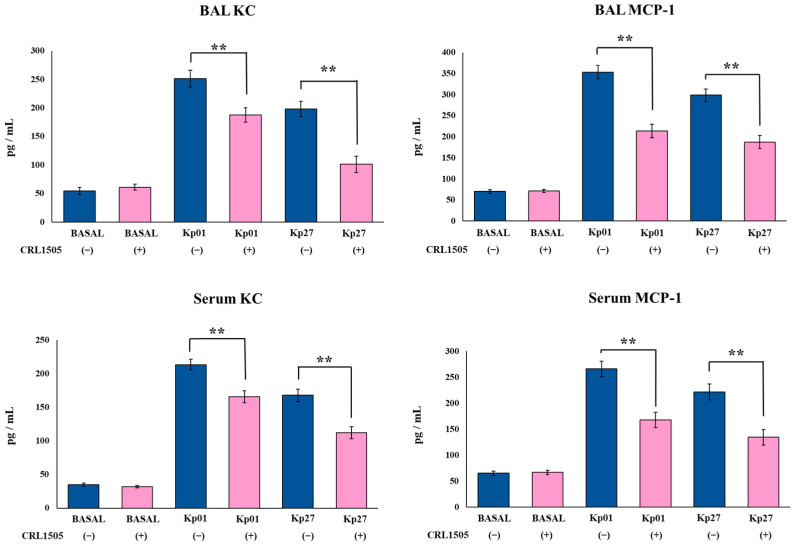
Effect of *Lacticaseibacillus rhamnosus* CRL1505 on respiratory and blood inflammatory chemokines induced by KPC-2-producing hypermucoviscous *Klebsiella pneumoniae* ST25 strains. BALB/c mice (6 weeks old) were orally treated with the CRL1505 strain for 5 days and then challenged nasally with *K. pneumoniae* LABACER 01 (Kp01) or LABACER 27 (Kp27). Before (basal) and two days after challenge, KC and MCP-1 levels were determined in broncho–alveolar lavages (BAL) and serum. Results represent data from three independent experiments. Asterisks indicate significant differences between the indicated groups, (**) *p* < 0.01. Pink bars represent mice given *Lcb. rhamnosus* CRL1505 and blue bars represent control mice.

The levels of the inflammatory cytokines TNF-α, IL-1β, and IL-6, as well as the inflammatory chemokines KC and MCP-1, were determined in BAL and serum samples before and after the challenge with the *K. pneumoniae* ST25 strains. The oral treatment of mice with the CRL1505 strain did not induce changes in the basal levels of BAL and serum TNF-α, IL-1β, IL-6 (Figure 3), KC and MPC-1 (Figure 4). The nasal challenge with LABACER 01 or LABACER 27 increased the levels of all the inflammatory factors compared to basal levels, in both control and lactobacilli-treated mice. However, the levels of BAL TNF-α, IL-1β, IL-6 (Figure 3), KC and MPC-1 (Figure 4) were lower in mice orally treated with *Lcb. rhamnosus* CRL1505 than in their respective control groups. The most remarkable effects were observed for BAL IL-1β and MCP-1. The levels of IL-1β in control mice infected with LABACER 01 and LABACER 27 strains were 324.5 ± 18.1 and 256.8 ± 19.2 pg/mL while in mice treated with the CRL1505 strain they were 234.23 ± 21.2 and 179.4 ± 20.3, respectively. The concentrations of MCP-1 in LABACER 01 and LABACER 27 control groups were 353.4 ± 15.8 and 298.6 ± 14.5 pg/mL, while in *Lcb. rhamnosus* CRL1505-treated mice they were 213.4 ± 16.2 and 187.5 ± 15.9, respectively. Similarly, serum TNF-α, IL-1β (Figure 3), KC and MPC-1 (Figure 4) were diminished in lactobacilli-treated mice compared to controls. Notably, no significant differences were found in serum IL-6 levels between mice receiving *Lcb. rhamnosus* CRL1505 and controls (Figure 3).

### 3.3. Lcb. rhamnosus CRL1505 Modulates Effector and Regulatory Cytokines Produced against K. pneumoniae ST25 Infection

**Figure 5:**
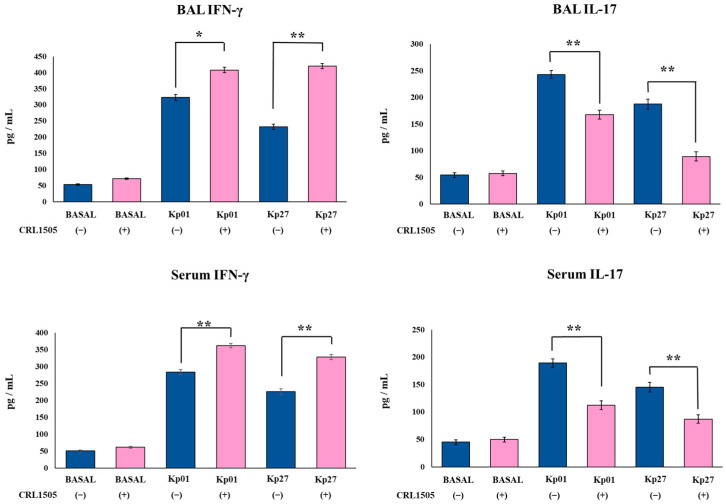
Effect of *Lacticaseibacillus rhamnosus* CRL1505 on respiratory and blood effector cytokines chemokines induced by KPC-2-producing hypermucoviscous *Klebsiella pneumoniae* ST25 strains. BALB/c mice (6 weeks old) were orally treated with the CRL1505 strain for 5 days and then challenged nasally with *K. pneumoniae* LABACER 01 (Kp01) or LABACER 27 (Kp27). Before (basal) and two days after challenge, IFN-γ and IL-17 levels were determined in broncho–alveolar lavages (BAL) and serum. Results represent data from three independent experiments. Asterisks indicate significant differences between the indicated groups, (*) *p* < 0.05, (**) *p* < 0.01. Pink bars represent mice given *Lcb. rhamnosus* CRL1505 and blue bars represent control mice.

**Figure 6:**
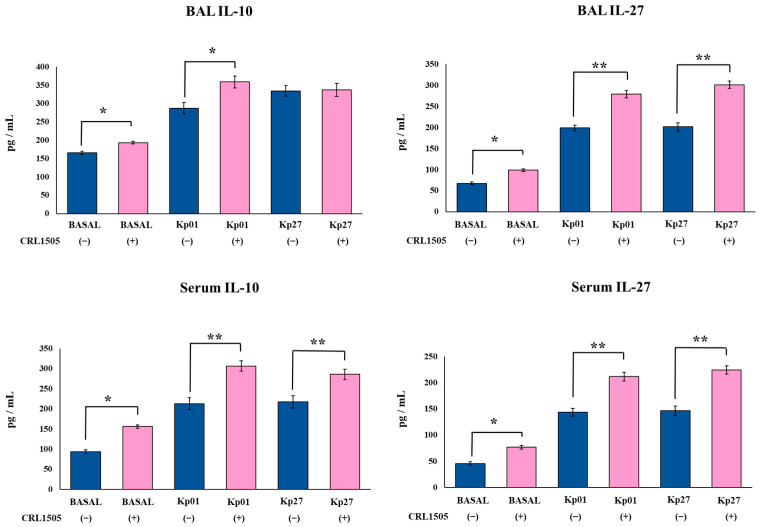
Effect of *Lacticaseibacillus rhamnosus* CRL1505 on respiratory and blood regulatory cytokines chemokines induced by KPC-2-producing hypermucoviscous *Klebsiella pneumoniae* ST25 strains. BALB/c mice (6 weeks old) were orally treated with the CRL1505 strain for 5 days and then challenged nasally with *K. pneumoniae* LABACER 01 (Kp01) or LABACER 27 (Kp27). Before (basal) and two days after challenge, IL-10 and IL-27 levels were determined in broncho–alveolar lavages (BAL) and serum. Results represent data from three independent experiments. Asterisks indicate significant differences between the indicated groups, (*) *p* < 0.05, (**) *p* < 0.01. Pink bars represent mice given *Lcb. rhamnosus* CRL1505 and blue bars represent control mice.

Finally, the levels of the effector cytokines IFN-γ and IL-17 as well as the regulatory cytokines IL-10 and IL-27 were evaluated in BAL and serum samples before and after the challenge with the LABACER strains. The oral treatment of mice with the CRL1505 strain did not induce changes in the basal levels of BAL and serum IFN-γ and IL-17 (Figure 5). However, the oral treatment of mice with *Lcb. rhamnosus* CRL1505 increased the levels of BAL and serum IL-10 and IL-27 in the steady state (Figure 6). The levels of BAL IL-27 and IL-10 in control mice were 67.4 ± 2.9 and 165.4 ± 4.2 pg/mL, respectively, while in mice treated with the CRL1505 strain they were 98.7 ± 3.1 and 193.3 ± 3.9. The nasal challenge with the *K. pneumoniae* ST25 strains significantly increased the levels of IFN-γ, IL-17, IL-10, and IL-27 in both the respiratory and systemic compartments in all the experimental groups. However, mice treated with the CRL1505 strain showed higher levels of BAL and serum IFN-γ (Figure 5), IL-10 and IL-27 (Figure 6) than controls. The levels of BAL IFN-γ in control mice infected with LABACER 01 and LABACER 27 strains were 323.9 ± 9.2 and 232.5 ± 8.3 pg/mL, while in mice treated with the CRL1505 strain they were 408.3 ± 8.6 and 420.3 ± 7.9, respectively. On the other hand, the concentrations of BAL IL-27 in LABACER 01 and LABACER 27 control groups were 198.6 ± 7.1 and 201.3 ± 9.2 pg/mL, while in *Lcb. rhamnosus* CRL1505-treated mice they were 278.9 ± 8.5 and 301.2 ± 8.7, respectively.

In contrast, mice treated with *Lcb. rhamnosus* CRL1505 had significantly lower levels of BAL and serum IL-17 than their respective control groups (Figure 5). In fact, the LABACER 01 and LABACER 27 control groups have values of BAL IL-17 of 243.4 ± 7.1 and 187.6 ± 9.2 pg/mL while mice receiving the CRL1505 strain showed values of 167.8 ± 8.2 and 89.5 ± 8.8, respectively.

## 4. Discussion

*K. pneumoniae* is one of the most prevalent pathogens causing nosocomial pneumonia [25]. The interest in the study of respiratory infections caused by this bacterium has been renewed in recent years because *K. pneumoniae* was identified as a major cause of secondary bacterial pneumonia in patients with COVID-19 [26]. Furthermore, the increase in hypervirulent *K. pneumoniae* strains capable of causing community-acquired infections in otherwise healthy hosts [27] and the enhanced antibiotic resistance among clinical isolates [28] necessitate more in-depth studies of the pathogenesis and immune response, as well as preventive and therapeutic alternatives for these bacteria. Murine models of respiratory infections caused by classical and hypervirulent *K. pneumoniae* strains have been useful in gaining deeper knowledge of the immunobiology of these infections [29,30]. In this regard, we have used mice as a model to characterize respiratory infections caused by multiresistant *K. pneumoniae* isolates from the ST25 considering that among the KPC-2-producing strains, this sequence type has emerged as a persistent and overrepresented cause of hospital-associated infections in the Northwest of Argentina [5,6,7]. We demonstrated that *K. pneumoniae* ST25 strains were able to infect the respiratory tract of adult immunocompetent mice after the nasal challenge, inducing a potent innate immune response characterized by the increase in inflammatory cytokines and chemokines and the recruitment of inflammatory cells into the lung [23,24]. In fact, our previous and present results demonstrated that the nasal challenge of mice with the ST25 *K. pneumoniae* strains LABACER 01 and LABACER 27 increased the levels of TNF-α, IL-1β, IL-6, IFN-γ, IL-17, KC and MPC-1 in the respiratory tract and blood, as well as the numbers of BAL neutrophils and macrophages. 

Inflammatory cytokines and chemokines have been shown to exert a protective role against respiratory *K. pneumoniae* infection. Deficiency or impairment of IFN-γ, IL-17 [31] and TNF-α [32] significantly reduce the resistance against *K. pneumoniae* infection. Recent transcriptomic studies performed in the lungs of mice infected with the clinical strain isolated from a patient with severe pneumonia, *K. pneumoniae* YBQ, demonstrated a remarkable activation of TNF and IL-17 signaling pathways and suggested that neutrophils and CCR2^+^ monocytes are the key to protection against the infection [33]. Similarly, a time course transcriptomic study of the lungs of mice infected with hypervirulent *K. pneumoniae* revealed a significant upregulation of inflammatory cytokines and chemokines genes [34]. Notably, the work suggested that the activation of TNF, IL-17, MAPK and NF-kB signaling pathways and the decrease in the expression of genes involved in the structural integrity of lung tissue may play key roles in the immunopathogenesis of *K. pneumoniae* infection. Then, acute inflammation in the lung is necessary to control *K. pneumoniae* infection but if it is not tightly regulated, it can cause damage to lung structures, impairing the lung’s normal functions.

Interestingly, studies performed in germ-free mice demonstrated that these animals had increased susceptibility to a lethal *K. pneumoniae* respiratory infection because of an impaired innate immune response [35]. This work highlighted the importance of the intestinal microbial population in regulating the respiratory immune response against this Gram-negative pathogen, as was previously described for Gram-positive [36] and viral [37,38] respiratory pathogens. Then, we aimed to evaluate whether the oral administration of an immunomodulatory probiotic strain could beneficially modulate the respiratory immunity and confer some degree of protection against LABACER 01 and LABACER 27 strains. Notably, we demonstrated here that the oral treatment of mice with *Lcb. rhamnosus* CRL1505 before the challenge with *K. pneumoniae* ST25 strains significantly improved the mice’s resistance to the pathogens. In our hands, CRL1505-treated mice had lower *K. pneumoniae* counts in lungs, decreased levels of lung injury markers, reduced levels of BAL macrophages and neutrophils and diminished concentrations of TNF-α, IL-1β, IL-6, IL-17, KC and MPC-1 in the respiratory tract and blood than infected controls. This is the first demonstration of the *Lcb. rhamnosus* CRL1505’s ability, when orally administered, to beneficially modulate the respiratory immune response against a Gram-negative bacterial pathogen. To the best of our knowledge, only two other studies have demonstrated the capacity of orally administered probiotics to beneficially modulate the immune response to *K. pneumoniae* respiratory infection. Experiments performed in adult C57BL/6 mice showed that the oral administration of *Bifidobacterium longum* 51A enhanced their resistance to the nasal challenge with *K. pneumoniae* ATCC 27,736 [16]. Animals treated with the 51A strain had improved survival and lower lung bacterial counts and injuries than infected controls. These benefits were associated with a differential regulation of the respiratory innate immune response, since *B. longum* 51A-treated mice had reduced levels of TNF-α, IL-6, CXCL1, and inflammatory cells in lungs [16]. Similarly, the oral administration of *Lactiplantibacillus plantarum* CIRM653 to C57BL/6 mice before the respiratory challenge with *K. pneumoniae* LM21 significantly enhanced the resistance to the pathogen by reducing BAL macrophages and neutrophils as well as the levels of BAL TNF-α, IL-6 and KC [15]. Moreover, in vitro studies showed that *L. plantarum* CIRM653 reduced the activation of the NF-κB pathway in airway epithelial cells induced by *K. pneumoniae* challenge, reducing the production of IL-8 and IL-6 [15]. 

In addition to the reduction in inflammatory cells and cytokines, orally administered *Lcb. rhamnosus* CRL1505 was able to increase the levels of the regulatory cytokines IL-10 and IL-27 in BAL and serum of the mice infected with LABACER 01 and LABACER 27 strains. In line with these results, we demonstrated that orally administered probiotics exert their beneficial effects against Gram-positive and viral respiratory pathogens by modulating the levels of the regulatory cytokines IL-10 and/or IL-27 [17,18,19,20,21,22]. It was also reported that the oral treatment of mice with *B. longum* 51A significantly increased the levels of BAL IL-10 during *K. pneumoniae* ATCC 27,736 infection [16]. Furthermore, it was shown that the respiratory challenge of mice with *K. pneumoniae* significantly increased the expression of *T-bet* in lungs, while no modifications were detected for *ROR-γt* or *foxp3* [15]. In contrast, mice treated with *L. plantarum* CIRM653 before *K. pneumoniae* challenge had reduced *T-bet* and increased *foxp3* and *il10* expression in the lung tissue [15]. In agreement, it was reported that the CIRM653 strain increased the numbers of CD4^+^CD25^+^Foxp3^+^ cells in the mediastinal lymph nodes of *K. pneumoniae*-infected mice. These and our own results suggest that the ability of probiotic microorganisms to help control detrimental inflammation in lungs during *K. pneumoniae* infection would be a key feature to improve the resistance to this pathogen.

It was shown that IL-10 inhibits immunopathological consequences induced by respiratory pathogens, including *K. pneumoniae*, particularly during the resolution phase of inflammation [39]. Although the beneficial role of IL-27 in regulating inflammation in the lungs has been described for several respiratory pathogens [40], there is a lack of studies directly investigating the effect of this regulatory cytokine in the context of *K. pneumoniae* respiratory infection. Considering the improved levels of respiratory and serum IL-27 in CRL1505-treated mice and the lower susceptibility of these mice to the lung bacterial colonization and damage, detailed evaluation of the role of IL-27 in *K. pneumoniae* infection, as well as in the protective effect of *Lcb. rhamnosus* CRL1505, is an interesting topic for future research. 

A range of recent studies, including from our group, have shown that some orally administered probiotic strains can exert beneficial effects in the immune responses of the respiratory tract and thus increase the protection against pathogens, particularly Gram-positive bacteria and viruses. In contrast, their potential beneficial effects against Gram-negative bacteria such as *K. pneumoniae* were less explored. In this work, we found a differential regulation of the respiratory and systemic immune responses triggered by the nasal infection with *K. pneumoniae* ST25 strains in mice preventively treated with *Lcb. rhamnosus* CRL1505 via the oral route. Although further analysis of immune factors and cells is required to clarify the mechanism involved in the beneficial effect of *Lcb. rhamnosus* CRL1505 during the infection with *K. pneumoniae*, this probiotic is an interesting candidate to improve protection of patients against hypermucoviscous KPC-2-producing strains belonging to the ST25, which is endemic in the hospitals of our region.

## Data Availability

All the data related to this project are presented here.

## References

[1] Gomez S.A., Pasteran F.G., Faccone D., Tijet N., Rapoport M., Lucero C., Lastovetska O., Albornoz E., Galas M., Melano R.G. (2011). Clonal dissemination of *Klebsiella pneumoniae* ST258 harbouring KPC-2 in Argentina. Clin. Microbiol. Infect..

[2] Cejas D., Fernandez Canigia L., Nastro M., Rodríguez C., Tanco A., Rodríguez H., Vay C., Maldonado I., Famiglietti A., Giovanakis M. (2012). Hyperendemic clone of KPC producing *Klebsiella pneumoniae* ST 258 in Buenos Aires hospitals. Infect. Genet. Evol..

[3] Cejas D., Elena A., Guevara Nuñez D., Sevilla Platero P., De Paulis A., Magariños F., Alfonso C., Berger M.A., Fernández-Canigia L., Gutkind G. (2019). Changing epidemiology of KPC-producing *Klebsiella pneumoniae* in Argentina: Emergence of hypermucoviscous ST25 and high-risk clone ST307. J. Glob. Antimicrob. Resist..

[4] Lin J., Huang Y., Qian L., Pan X., Song Y. (2022). Liver Abscess Combined with Endogenous Endophthalmitis Caused by Genotype ST25 Serotype K2 Hypervirulent *Klebsiella pneumoniae*: A Case Report. Infect. Drug Resist..

[5] Vargas J.M., Moreno Mochi M.P., Nuñez J.M., Cáceres M., Mochi S., del Campo Moreno R., Jure M.A. (2019). Virulence factors and clinical patterns of multiple-clone hypermucoviscous KPC-2 producing K. pneumoniae. Heliyon.

[6] Jure M.A., Castillo M.E., Musa H.E., López C.G., Cáceres M.A., Mochi S.D., Bousquet A.A., Genel N.A., Arlet G.A., Decré D.C. (2019). Novel patterns in the molecular epidemiology of KPC-producing *Klebsiella pneumoniae* in Tucumán, Argentina. J. Glob. Antimicrob. Resist..

[7] Jure M.A., Albarracin L., Vargas J.M., Maidana S.D., Zamar J.C., Kitazawa H., Villena J. (2021). Draft genome sequences of two hypermucoviscous carbapenem-resistant ST25 *Klebsiella pneumoniae* strains causing respiratory and systemic infections. J. Glob. Antimicrob. Resist..

[8] Villena J., Kitazawa H. (2020). The Modulation of Mucosal Antiviral Immunity by Immunobiotics: Could They Offer Any Benefit in the SARS-CoV-2 Pandemic?. Front. Physiol..

[9] Raras T.Y.M., Firdausy A.F., Kinanti I.R., Noorhamdani N. (2019). Anti-Biofilm Activity of Lactic Acid Bacteria Isolated from Kefir against Multidrug-Resistant *Klebsiella pneumoniae*. J. Pure Appl. Microbiol..

[10] El-Mokhtar M.A., Hassanein K.M., Ahmed A.S., Gad G.F.M., Amin M.M., Hassanein O.F.E. (2020). Antagonistic Activities of Cell-Free Supernatants of Lactobacilli Against Extended-Spectrum &beta;-Lactamase Producing *Klebsiella pneumoniae* and *Pseudomonas aeruginosa*. Infect. Drug Resist..

[11] Yan R., Lu Y., Wu X., Yu P., Lan P., Wu X., Jiang Y., Li Q., Pi X., Liu W. (2021). Anticolonization of Carbapenem-Resistant *Klebsiella pneumoniae* by *Lactobacillus plantarum* LP1812 through Accumulated Acetic Acid in Mice Intestinal. Front. Cell. Infect. Microbiol..

[12] Lagrafeuille R., Miquel S., Balestrino D., Vareille-Delarbre M., Chain F., Langella P., Forestier C. (2017). Opposing effect of *Lactobacillus* on in vitro *Klebsiella pneumoniae* in biofilm and in an in vivo intestinal colonisation model. Benef. Microbes.

[13] Ermolenko E., Rybalchenko O., Borshev Y., Tarasova E., Kramskaya T., Leontieva G., Kotyleva M., Orlova O., Abdurasulova I., Suvorov A. (2018). Influence of monostrain and multistrain probiotics on immunity, intestinal ultrastructure and microbiota in experimental dysbiosis. Benef. Microbes.

[14] Savinova O.S., Glazunova O.A., Moiseenko K.V., Begunova A.V., Rozhkova I.V., Fedorova T.V. (2021). Exoproteome analysis of antagonistic interactions between the probiotic bacteria *Limosilactobacillus reuteri* LR1 and *Lacticaseibacillus rhamnosus* F and multidrug resistant strain of klebsiella pneumonia. Int. J. Mol. Sci..

[15] Vareille-Delarbre M., Miquel S., Garcin S., Bertran T., Balestrino D., Evrard B., Forestier C. (2019). Immunomodulatory effects of *Lactobacillus plantarum* on inflammatory response induced by *Klebsiella pneumoniae*. Infect. Immun..

[16] Vieira A.T., Rocha V.M., Tavares L., Garcia C.C., Teixeira M.M., Oliveira S.C., Cassali G.D., Gamba C., Martins F.S., Nicoli J.R. (2016). Control of *Klebsiella pneumoniae* pulmonary infection and immunomodulation by oral treatment with the commensal probiotic *Bifidobacterium longum* 51A. Microbes Infect..

[17] Raya Tonetti F., Clua P., Fukuyama K., Marcial G., Sacur J., Marranzino G., Tomokiyo M., Vizoso-Pinto G., Garcia-Cancino A., Kurata S. (2022). The ability of postimmunobiotics from *L. rhamnosus* CRL1505 to Protect against respiratory syncytial virus and pneumococcal super-infection is a strain-dependent characteristic. Microorganisms.

[18] Kitazawa H., Villena J. (2014). Modulation of respiratory TLR3-anti-viral response by probiotic microorganisms: Lessons learned from *Lactobacillus rhamnosus* CRL1505. Front. Immunol..

[19] Garcia-Castillo V., Tomokiyo M., Raya Tonetti F., Islam M.A., Takahashi H., Kitazawa H., Villena J. (2020). Alveolar Macrophages Are Key Players in the Modulation of the Respiratory Antiviral Immunity Induced by Orally Administered *Lacticaseibacillus rhamnosus* CRL1505. Front. Immunol..

[20] Albarracin L., Garcia-Castillo V., Masumizu Y., Indo Y., Islam M.A., Suda Y., Garcia-Cancino A., Aso H., Takahashi H., Kitazawa H. (2020). Efficient Selection of New Immunobiotic Strains With Antiviral Effects in Local and Distal Mucosal Sites by Using Porcine Intestinal Epitheliocytes. Front. Immunol..

[21] Chiba E., Tomosada Y., Vizoso-Pinto M.G., Salva S., Takahashi T., Tsukida K., Kitazawa H., Alvarez S., Villena J. (2013). Immunobiotic *Lactobacillus rhamnosus* improves resistance of infant mice against respiratory syncytial virus infection. Int. Immunopharmacol..

[22] Salva S., Villena J., Alvarez S. (2010). Immunomodulatory activity of *Lactobacillus rhamnosus* strains isolated from goat milk: Impact on intestinal and respiratory infections. Int. J. Food Microbiol..

[23] Dentice Maidana S., Ortiz Moyano R., Vargas J.M., Fukuyama K., Kurata S., Melnikov V., Jure M.Á., Kitazawa H., Villena J. (2022). Respiratory Commensal Bacteria Increase Protection against Hypermucoviscous Carbapenem-Resistant *Klebsiella pneumoniae* ST25 Infection. Pathogens.

[24] Albarracin L., Ortiz Moyano R., Vargas J.M., Andrade B.G.N., Zamar J.C., Dentice Maidana S., Fukuyama K., Kurata S., Jure M.Á., Kitazawa H. (2022). Genomic and Immunological Characterization of Hypermucoviscous Carbapenem-Resistant *Klebsiella pneumoniae* ST25 Isolates from Northwest Argentina. Int. J. Mol. Sci..

[25] Lou W., Venkataraman S., Zhong G., Ding B., Tan J.P.K., Xu L., Fan W., Yang Y.Y. (2018). Antimicrobial polymers as therapeutics for treatment of multidrug-resistant *Klebsiella pneumoniae* lung infection. Acta Biomater..

[26] Zhu X., Ge Y., Wu T., Zhao K., Chen Y., Wu B., Zhu F., Zhu B., Cui L. (2020). Co-infection with respiratory pathogens among COVID-2019 cases. Virus Res..

[27] Russo T.A., Marr C.M. (2019). Hypervirulent *Klebsiella pneumoniae*. Clin. Microbiol. Rev..

[28] CDCP (2019). Antibiotic Resistance Threats in the United States, 2019.

[29] Wasbotten R.K., Dahler A.A., Mackel J.J., Smith C.M., Rosen D.A. (2022). Murine Respiratory Tract Infection with Classical *Klebsiella pneumoniae* Induces Bronchus-Associated Lymphoid Tissue. Infect. Immun..

[30] Ahn D., Bhushan G., McConville T.H., Annavajhala M.K., Soni R.K., Wong Fok Lung T., Hofstaedter C.E., Shah S.S., Chong A.M., Castano V.G. (2021). An acquired acyltransferase promotes *Klebsiella pneumoniae* ST258 respiratory infection. Cell Rep..

[31] Happel K.I., Dubin P.J., Zheng M., Ghilardi N., Lockhart C., Quinton L.J., Odden A.R., Shellito J.E., Bagby G.J., Nelson S. (2005). Divergent roles of IL-23 and IL-12 in host defense against *Klebsiella pneumoniae*. J. Exp. Med..

[32] Moore T.A., Lau H.Y., Cogen A.L., Standiford T.J. (2005). Defective innate antibacterial host responses during murine *Klebsiella pneumoniae* bacteremia: Tumor necrosis factor (TNF) receptor 1 deficiency versus therapy with anti-TNF-alpha. Clin. Infect. Dis..

[33] Lei L., Zhang X., Yang R., Jing H., Yuan Y., Chen Z., Gou Q., Zhao Z., Zhang J., Wang X. (2022). Host Immune Response to Clinical Hypervirulent *Klebsiella pneumoniae* Pulmonary Infections via Transcriptome Analysis. J. Immunol. Res..

[34] Zheng X., Guo J., Cao C., Qin T., Zhao Y., Song X., Lv M., Hu L., Zhang L., Zhou D. (2022). Time-Course Transcriptome Analysis of Lungs From Mice Infected With Hypervirulent *Klebsiella pneumoniae* via Aerosolized Intratracheal Inoculation. Front. Cell. Infect. Microbiol..

[35] Fagundes C.T., Amaral F.A., Vieira A.T., Soares A.C., Pinho V., Nicoli J.R., Vieira L.Q., Teixeira M.M., Souza D.G. (2012). Transient TLR activation restores inflammatory response and ability to control pulmonary bacterial infection in germfree mice. J. Immunol..

[36] Schuijt T.J., Lankelma J.M., Scicluna B.P., De Sousa E Melo F., Roelofs J.J.T.H., De Boer J.D., Hoogendijk A.J., De Beer R., De Vos A., Belzer C. (2016). The gut microbiota plays a protective role in the host defence against pneumococcal pneumonia. Gut.

[37] Ichinohe T., Pang I.K., Kumamoto Y., Peaper D.R., Ho J.H., Murray T.S., Iwasaki A. (2011). Microbiota regulates immune defense against respiratory tract influenza a virus infection. Proc. Natl. Acad. Sci. USA.

[38] Abt M.C., Osborne L.C., Monticelli L.A., Doering T.A., Alenghat T., Sonnenberg G.F., Paley M.A., Antenus M., Williams K.L., Erikson J. (2012). Commensal bacteria calibrate the activation threshold of innate antiviral immunity. Immunity.

[39] Peñaloza H.F., Noguera L.P., Ahn D., Vallejos O.P., Castellanos R.M., Vazquez Y., Salazar-Echegarai F.J., González L., Suazo I., Pardo-Roa C. (2019). Interleukin-10 produced by Myeloid-Derived suppressor cells provides protection to Carbapenem-Resistant *Klebsiella pneumoniae* sequence type 258 by enhancing its clearance in the airways. Infect. Immun..

[40] Branchett W.J., Lloyd C.M. (2019). Regulatory cytokine function in the respiratory tract. Mucosal Immunol..

